# SPOP–PTEN–SUFU axis promotes progression of clear cell renal cell carcinoma via activating SHH and WNT pathway

**DOI:** 10.1038/s41420-021-00484-2

**Published:** 2021-05-21

**Authors:** Bo’ang Han, Zhen Sun, Tingting Yu, Yu Wang, Lun Kuang, Tianyuan Li, Jing Cai, Qing Cao, Yuan Xu, Binbin Gao, Steven Y. Cheng, Shen Yue, Chen Liu

**Affiliations:** 1 Department of Medical Genetics, Nanjing Medical University, 211166 Nanjing, China; 2 Jiangsu Key Lab of Cancer Biomarkers, Prevention and Treatment, Collaborative Innovation Center for Cancer Personalized Medicine, Nanjing Medical University, 211166 Nanjing, China; 3 Jiangsu Key Laboratory of Xenotransplantation, Nanjing Medical University, 211166 Nanjing, China; 4 Department of Pathology, Affiliated Zhongshan Hospital of Dalian University, 116001 Dalian, China; 5 College of Medicine, Henan University of Science and Technology, 471023 Luoyang, China; 6 The First School of Clinical Medicine, Nanjing Medical University, 211166 Nanjing, China

**Keywords:** Renal cell carcinoma, Tumour biomarkers

## Abstract

Although E3 ligase Speckle type BTB/POZ protein (SPOP) promotes tumorigenesis by acting as a key regulatory hub in clear cell renal cell carcinoma (ccRCC), the detailed molecular mechanism remains unclear. Here, we demonstrate that a well-known tumor suppressor, Suppressor of Fused (SUFU), is downregulated by SPOP. Interestingly, this downregulation depends on cullin-3(Cul3)-SPOP E3 ligase, but SUFU is not a direct substrate of SPOP. Phosphatase and tensin homolog (PTEN), a ubiquitinated substrate of SPOP, is involved in SPOP-mediated SUFU reduction. Importantly, inhibition of SUFU leads to elevated SHH and WNT signaling, consequently rescuing the reduced proliferation, migration, and invasion abilities of ccRCC cells caused by SPOP-knockdown. Moreover, combinatorial treatment with SHH and WNT inhibitors shows more effective for suppressing ccRCC cell proliferation and aggressiveness. These findings demonstrate that a novel SPOP–PTEN–SUFU axis promotes ccRCC carcinogenesis by activating SHH and WNT pathway, providing a new treatment strategy for ccRCC.

## Introduction

Renal cell carcinoma (RCC) is a common malignant tumor of the urinary system. There are >73,000 new patients every year in the United States, and >14,000 people die of this disease, with a growing trend in 2020 (ref. ^1^). Clear cell renal cell carcinoma (ccRCC) is the most common pathological subtype of RCC, accounting for ~75% of all cases^2^. In 30% of patients, cancer cells have already metastasized at the time of diagnosis, and 50% of the remaining patients will also undergo metastasis of cancer cells. Metastatic RCC is usually difficult to cure, with a 5-year survival rate of <10% (ref. ^3^). Thus, the molecular mechanisms involved in ccRCC development and promising therapeutic approaches for this fatal disease are urgently needed to be explored.

The ubiquitin E3 ligase speckle type BTB/POZ protein (SPOP), acting as a key regulatory hub in kidney cancer, has been reported to promote ccRCC tumorigenesis and progression through ubiquitinating and degrading several tumor suppressors^4,5^. Almost in all ccRCCs, SPOP is overexpressed and accumulated in the cytoplasm under hypoxia^5^. Overexpressed SPOP in ccRCC cells promotes proliferation and inhibits apoptosis through the ubiquitin–proteasome-mediated degradation of phosphatase and tensin homolog (PTEN), dual-specificity phosphatase 7 (DUSP7), death domain-associated protein (DAXX), and Sonic hedgehog (SHH) transcription factor glioma-associated oncogene homolog 2 (GLI2). Meanwhile, SPOP promotes the tumor invasiveness by degrading large tumor suppressor 1(LAST1) or enhancing the transcription factor β-catenin protein expression, as well as its nuclear translocation in WNT signaling pathway^5–7^. In these studies, although SPOP was shown to promote ccRCC tumorigenesis and progression, and small molecule compound 6b has been indicated to inhibit this process, the downstream signaling pathway controlled by SPOP is still unclear, which may be critical for an understanding of ccRCC pathogenesis^8^. Thus, further investigation of the mechanism involving SPOP in ccRCC is required.

In our previous study, Suppressor of Fused (SUFU) is reported to be downregulated by Hedgehog-induced BTB protein (HIB), which is the homolog of SPOP in *Drosophila*. Moreover, mammalian SPOP can substitute for HIB to suppress SUFU in flies, suggesting its conserved role in regulation of SUFU^9,10^. SUFU is generally accepted as a repressor in SHH signaling pathway, playing vital roles in the production, trafficking, and function of three transcription factors GLI1, GLI2, and GLI3 (ref. ^11–14^). In addition, as a cross-linking point of SHH and WNT pathways, SUFU also promotes the nuclear export process of the transcriptional activator β-CATENIN to negatively regulate WNT signaling transduction^15,16^. In humans, the loss of SUFU function has been shown to be associated with the tumorigenesis and progression in many cancers, such as medulloblastoma, basal cell carcinoma, and rhabdomyoma, indicating that SUFU is a tumor suppressor gene^17–19^. However, the specific function of SUFU in ccRCC is unknown. All of the above prompted us to investigate whether SPOP promotes tumorigenesis and progression in ccRCC by modulating SUFU repressor activity in SHH and WNT pathway.

## Results

### SPOP downregulates SUFU in mammals

**Fig. 1:**
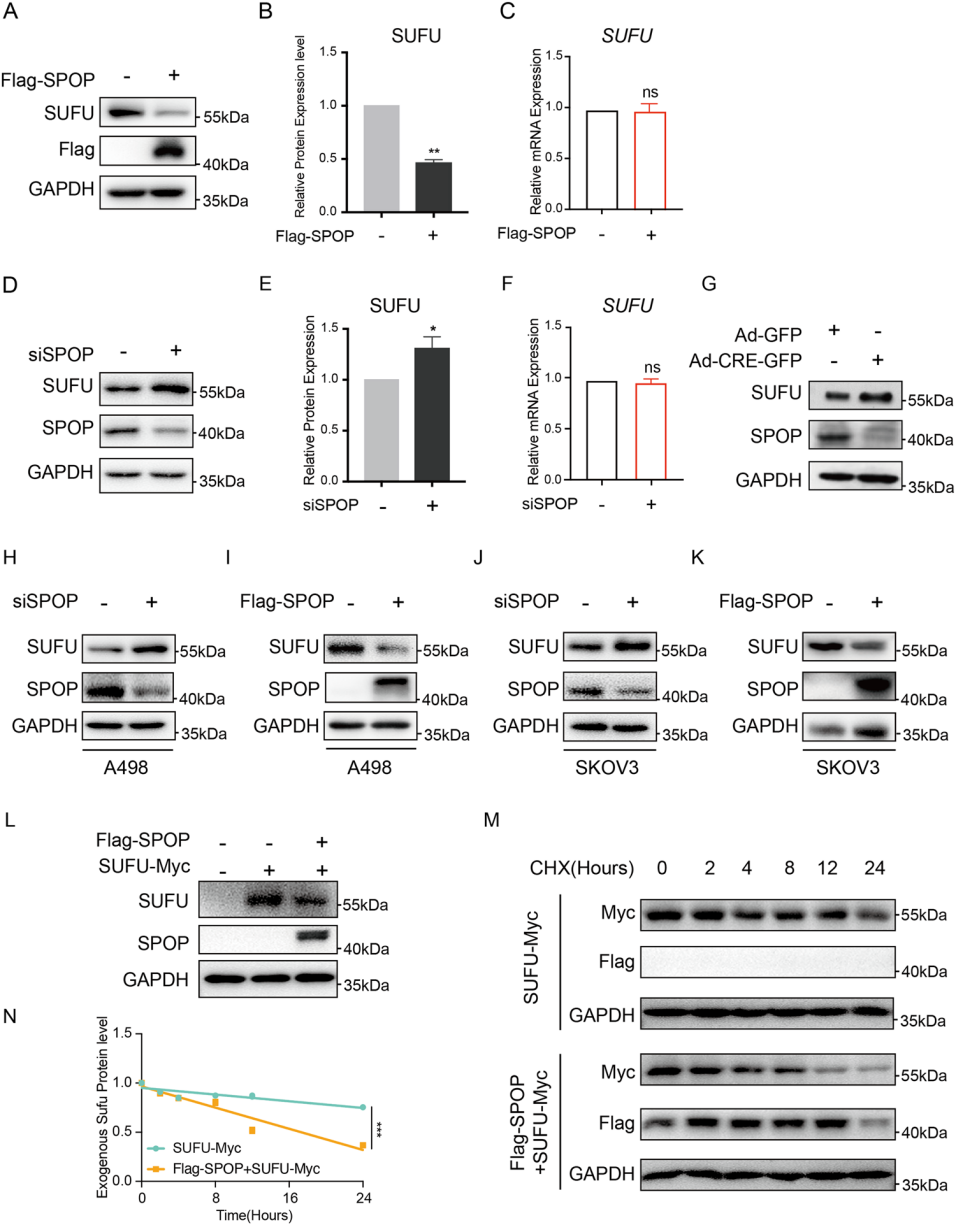
SPOP negatively regulates SUFU in mammals. **A**–**F** After transfection of indicated plasmids or siRNAs into 293T cells, SPOP and SUFU levels were verified by western blotting and qRT-PCR, and the abundance of SUFU protein in **A** and **D** was quantified. **G** SUFU protein levels were detected by western blot analysis of SPOP^foxl/flox^ cells infected with Ad-Cre-GFP viruses in comparison with cells infected with Ad-GFP viruses. **H**–**K** Protein levels of SUFU were determined by western blot analysis of the kidney cancer cell line A498 and ovarian cancer cell line SKOV3 after indicated transfection. **L** SUFU-Myc protein levels were examined by western blot analysis of 293T cells which were transfected with Flag-SPOP. Western blot analysis **M** and quantification **N** showed stabilization of SUFU-Myc in 293T cells transfected with RK5-Flag-SPOP. Protein synthesis was blocked with cycloheximide (CHX) treatment. The error bars indicate SD. *P < 0.05; **P < 0.01; ***P < 0.001; ns not significant (unpaired Student’s *t* test).

Our previous study reported that SUFU is downregulated by HIB/SPOP in *Drosophila*, so we further evaluated whether SUFU can be suppressed by SPOP in mammalian system^9^. First, ectopic expression of SPOP significantly reduced the protein level of endogenous SUFU in human embryonic kidney 293T cells (Fig. 1A, B). To exclude the possibility that the reduction was due to transcriptional downregulation, we performed quantitative real-time PCR (qRT-PCR) to measure the mRNA levels of SUFU in SPOP-overexpressing cells. In contrast to the significant increase in *SPOP* mRNA levels, *SUFU* mRNA in 293T cells ectopically expressing SPOP remained similar to that of the control cells, indicating that the effect of SPOP on SUFU was not mediated by the downregulation of *SUFU* mRNA (Fig. 1C). Consistently, knockdown of *SPOP* by small interfering RNA (siRNA) drastically upregulated endogenous SUFU protein level, while its mRNA level remained unchanged (Fig. 1D–F). To further confirm that SPOP negatively regulated SUFU, we used *Spop*-knockout MEFs. The data showed that the expression of endogenous SUFU was significantly elevated in *Spop*-knockout MEFs, confirming that SPOP downregulated SUFU in mammals (Fig. 1G). Furthermore, ectopic expression of SPOP decreased SUFU protein levels in the kidney cancer cell line A498 and ovarian cancer cell line SKOV3, and *siSPOP* showed the opposite results, suggesting the physiological significance of this regulation in carcinogenesis (Fig. 1H–K). In addition, co-expression of RK5-Flag-SPOP with RK5-SUFU-Myc reduced the level of exogenously expressed SUFU detected by anti-Myc antibody staining (Fig. 1L). To further determine whether SPOP regulates SUFU protein stability, we transfected 293T cells with RK5-Flag-SPOP and treated cells with cycloheximide (CHX) to block protein synthesis. The results showed that the turnover of exogenous SUFU in SPOP-overexpressed cells was markedly faster than that in control cells, with a half-life shortened from >24 to 12 h, suggesting that SUFU was affected by SPOP at posttranslational stage (Fig. 1M, N).

### Regulation of SUFU depends on CUL3-SPOP E3 ligase activity

**Fig. 2:**
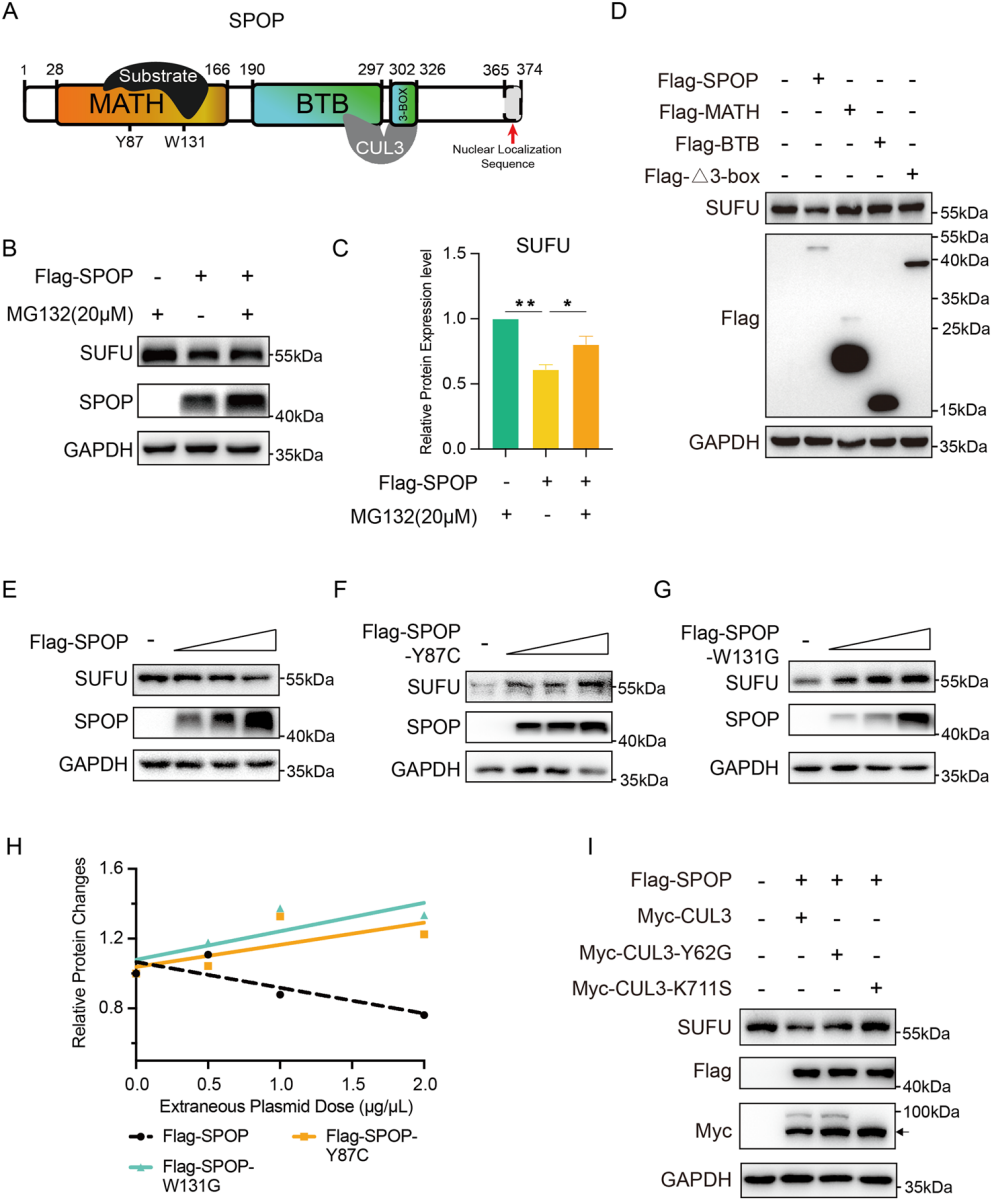
Cul3-SPOP E3 ligase activity is required for SUFU downregulation. **A** Schematic diagram shows SPOP interacting with substrates through MATH domain and with Cul3 through BTB/3-box domain. **B** Western blot analysis of SUFU derived from 293T cells transfected with indicated constructs. Cells were treated with MG132 before harvest. **C** The experiments in **B** were performed at least three times, and the abundance of SUFU protein was quantified. **D** SUFU protein levels were examined by western blot analysis of 293T cells, which were transfected with Flag-SPOP, Flag-MATH, Flag-BTB, or Flag-Δ3-box, respectively. **E**–**G** 293T cells were transfected with wild-type SPOP or Y87C and W131G mutants. The changes in SUFU protein expression were measured by western blotting. **H** The abundance of SUFU protein in **E**–**G** was quantified and plotted. **I** SUFU levels were examined in 293T cells co-expressing SPOP and wild-type CUL3 or Y62G and K711S mutants by western blot analysis. *P < 0.05; **P < 0.01 (unpaired Student’s *t* test).

As an E3 ligase, SPOP generally forms a complex with CUL3 through BTB/3-box domain and with substrates through the MATH domain, promoting the ubiquitin/proteasome-mediated degradation of targets (Fig. 2A)^20^. To address whether the destabilization of SUFU protein depends on CUL3-SPOP E3 ligase activity, we first employed the proteasome inhibitor MG132 and found it could partially block SPOP-mediated SUFU downregulation (Fig. 2B, C). Moreover, overexpression of full-length SPOP, but not SPOP MATH, BTB, or Δ3-box (lacking 3-box motif) variants, resulted in a reduction of SUFU protein level, indicating that the MATH, BTB, and 3-box domains are all required for SPOP-mediated SUFU downregulation (Fig. 2D). This result was expected because the C-terminal BTB/3-box domain of SPOP is necessary for its interaction with the scaffold protein CUL3, and the N-terminal MATH domain is essential for its interaction with SPOP-targeted substrate involved in the SUFU decrease. To avoid dramatic conformational changes in these truncated SPOP mutants caused by the loss of large peptide fragments, we applied two prostate cancer-associated SPOP point mutations, SPOP-Y87C (Tyr 87 replaced by Cys) and SPOP-W131G (Trp 131 replaced by Gly), which play dominant-negative roles in substrate binding and degradation^21,22^. The results showed that two SPOP mutants were no longer able to reduce SUFU protein and even stabilized it compared to wild-type SPOP, possibly by neutralizing endogenous SPOP (Fig. 2E–H). In addition, it has been reported that CUL3 mutants CUL3-Y62G (Tyr 62 replaced by Gly) and CUL3-K711S (Lys 711 replaced by Ser) cannot interact with SPOP and are modified by neural precursor cell expressed, developmentally downregulated 8 (NEDD8), respectively, which could affect SPOP-targeted substrates degradation through the ubiquitin–proteasome pathway^23,24^. To test whether the E3 ligase activity of SPOP-CUL3 is essential for SUFU reduction, we co-expressed SPOP with each of these two CUL3 mutants, respectively. Compared with wild-type Cul3, Y62G, and K711S mutants stabilized SUFU in 293T cells (Fig. 2I). Collectively, these data suggest that Cul3-SPOP E3 ligase activity is involved in SUFU decrease.

### SPOP negatively regulates SUFU through PTEN

**Fig. 3:**
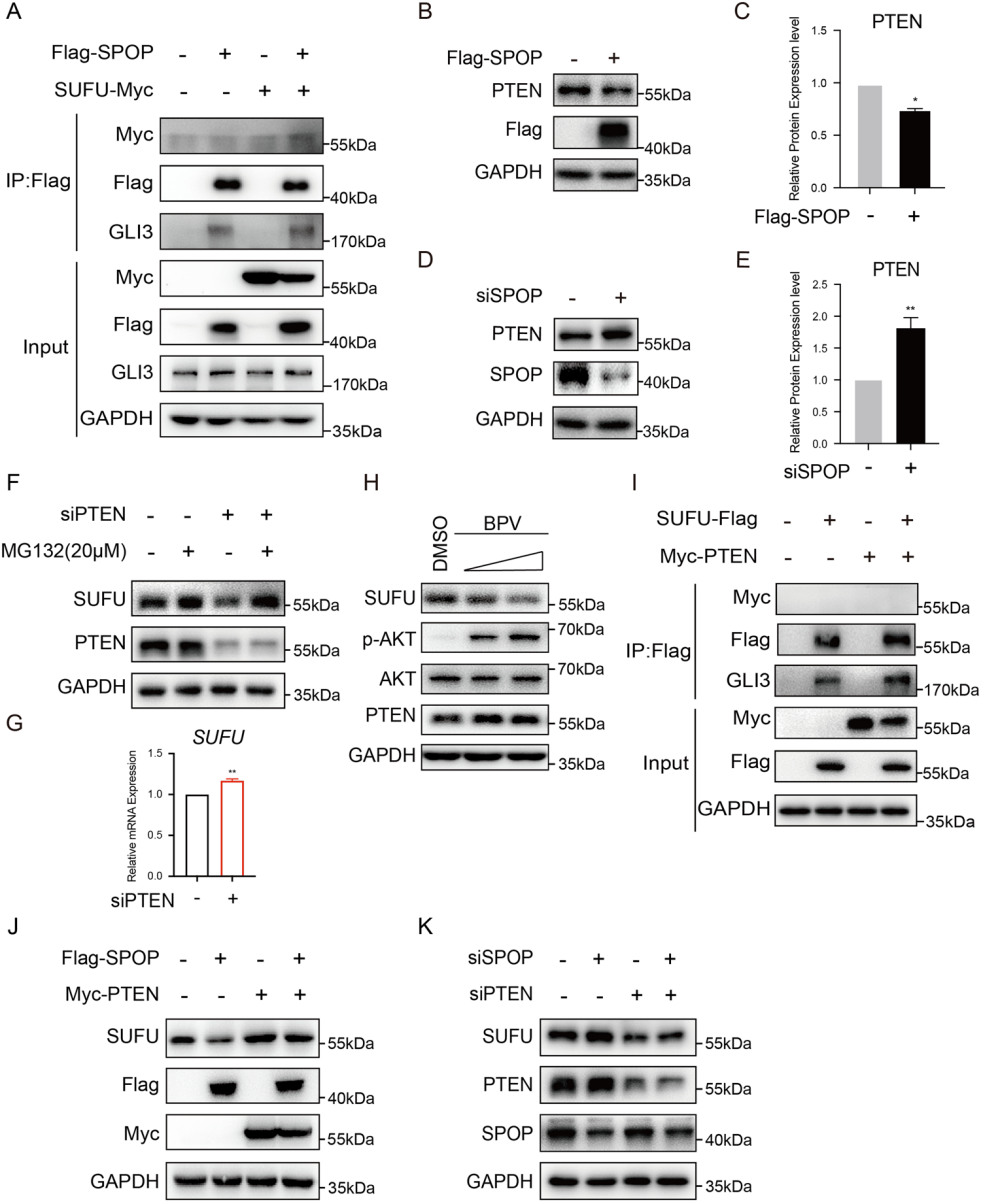
PTEN is involved in SPOP-induced downregulation of SUFU. **A** The binding between SPOP and SUFU was measured by Co-IP experiments. GLI3, which is a substrate of SPOP, was used as a positive control. **B**, **C** Western blot analysis and quantification of PTEN expression in 293T cells transfected with Flag-SPOP was performed. **D**, **E** Protein levels of PTEN were determined by western blot analysis of 293T cells transfected with *SPOP* siRNA or the corresponding nontargeted control, and the abundance of PTEN protein in **D** was quantified. **F**, **G** Protein and mRNA levels of SUFU were examined by western blotting and qRT-PCR, respectively, in 293T cells treated with PTEN siRNA. Cells were treated with MG132 before harvest. **H** The levels of SUFU were measured by western blot analysis of 293T cells treated with PTEN inhibitor BPV. **I** The binding between PTEN and SUFU was measured by Co-IP experiments. **J** 293T cells were transfected with Flag-SPOP, Myc-PTEN, or both constructs. Immunoblots of SUFU in transfected 293T cells are shown. **K** The change in indicated protein levels in 293T cells were analyzed via immunoblotting with anti-SUFU, anti-PTEN, and anti-SPOP antibodies as indicated. Immunoblot analysis of GAPDH was performed to confirm equivalent protein loading.

In Cul3-SPOP E3 ligase complex, SPOP specifically recognizes substrates for ubiquitination and degradation through direct binding. To investigate whether SPOP forms a complex with SUFU, we co-expressed SPOP and SUFU in 293T cells. Coimmunoprecipitation (Co-IP) data showed that SPOP failed to bind to SUFU, but it bound to GLI3, which had been proven to be a substrate of SPOP (Fig. 3A). Previous studies have demonstrated that most SPOP substrates share a conserved SPOP-binding consensus motif (φ-π-S-S/T-S/T; φ is nonpolar, π is polar)^20^. Accordingly, we analyzed SUFU protein sequence and found that SUFU did not contain such a motif. These findings suggested that SUFU was not a direct ubiquitinated substrate of SPOP.

These data collectively indicated that SUFU reduction might be indirectly regulated by CUL3-SPOP E3 ligase, therefore raising the possibility that this process is mediated through a certain substrate of SPOP. Coincidentally, we found that SUFU protein level was decreased in MEFs treated with PTEN inhibitor BPV (data not shown). Moreover, a recent study revealed that PTEN is a substrate for SPOP-mediated ubiquitination and degradation^5^. These results offered an opportunity to investigate whether SPOP downregulates SUFU through PTEN. To test this hypothesis, we first detected PTEN protein levels in 293T cells ectopically expressing SPOP or knocking down SPOP. Western blot results showed that the protein levels of PTEN were reduced in SPOP-overexpressed cells and increased in SPOP-knockdown cells (Fig. 3B–E). To verify the effect of PTEN on SUFU, we examined the SUFU levels after the treatment with *PTEN* siRNA. The results demonstrated that SUFU protein level was decreased in *siPTEN-*treated cells, which could be completely reversed by addition of MG132, while the mRNA level of SUFU was slightly increased (Fig. 3F, G). PTEN inhibited by its inhibitor BPV was additionally performed in 293T cells with similar results to *PTEN* siRNA (Fig. 3H). As a dual-specificity phosphatase (DUSP), PTEN is able to target serine, threonine, and tyrosine residues^25^. Therefore, we first performed Co-IP experiments to examine if PTEN formed a complex with SUFU. The results showed that PTEN did not interact with SUFU, suggesting that SUFU is not a protein substrate for PTEN (Fig. 3I). Notably, to verify whether PTEN was involved in the SPOP-mediated downregulation of SUFU, we examined SUFU protein levels after overexpressing or knocking down both SPOP and PTEN. As shown in Fig. 3J, overexpressing PTEN substantially abrogated the ability of SPOP to negatively regulate SUFU in 293T cells. Consistently, knocking down *PTEN* also exhibited significantly reduced SUFU protein levels with or without SPOP expression (Fig. 3K). These data collectively supported the conclusion that PTEN participates in SPOP-induced downregulation of SUFU.

### The expression of SPOP and SUFU is negatively correlated in ccRCC

**Fig. 4:**
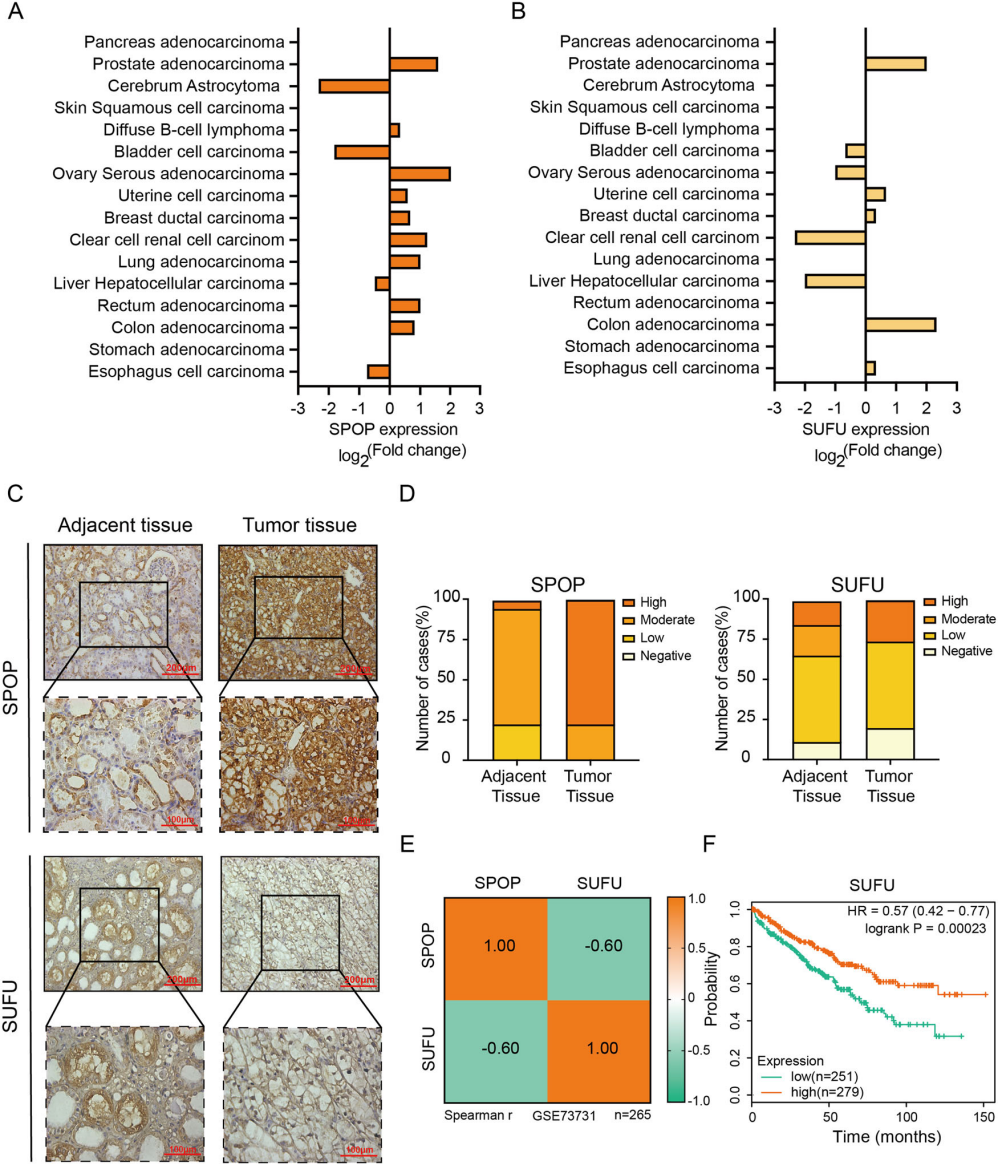
SPOP is negatively correlated with SUFU in ccRCC. **A**, **B** The statistical results for IHC staining of SPOP and SUFU among 16 different cancer types in human tissue microarrays. **C**, **D** Representative IHC staining for SPOP and SUFU in ccRCC tissues and normal adjacent tissues. **E** Spearman correlation analysis indicated that the mRNA level of SUFU was also negatively correlated with SPOP in ccRCC in GEO database (GSE73731). **F** Kaplan–Meier survival analysis of overall survival rate of ccRCC patients with high or low SUFU mRNA expression performed by using a dataset from Kaplan–Meier plotter.

To explore the potential clinical significance of SUFU downregulation by SPOP, we first performed the immunohistochemical staining to evaluate the levels of SPOP and SUFU among 16 different types of cancers, using human tumor tissue microarrays (Fig. 4A, B). The statistical results revealed that the expression of SPOP was significantly higher in ccRCC and ovarian serous adenocarcinoma tissues than in the corresponding adjacent normal tissues, while the distribution of SUFU was the opposite. SPOP has been demonstrated to be highly expressed in 99% of ccRCC tissues, in which it promotes tumorigenesis by degrading several substrates, including PTEN^5^. In addition, PTEN, which exhibits low expression in tumor tissues, could reduce the survival of ccRCC cells. Considering these reasons, we set our sight on ccRCC in the follow-up study. Although the results from three ccRCC tissues were the same in the above multi-tumor tissue assay, more ccRCC samples were needed for further investigation. Therefore, we examined 46 ccRCC and their adjacent normal tissues to confirm the negative correlation between SPOP and SUFU. SPOP and SUFU expression levels were categorized according to their staining intensity as: negative, low, moderate, and high (Supplementary Fig. S1A). IHC staining revealed that 15.22% of normal adjacent tissues showed high immunoreactivity for SUFU, 19.56% moderate, 54.35% low, and 10.87% negative. Conversely, negative immunoreactivity for SUFU was present in 19.57% of ccRCC tissues, low in 54.34%, moderate in 26.09%, and no high immunoreactivity for SUFU, illustrating a decrease of SUFU expression in cancerous tissues. The statistical results for SPOP agreed with those in the previous studies^4^. Overall, in ccRCC tissues, SPOP is overexpressed while SUFU is reduced (Fig. 4C, D). We also examined three pairs of ccRCC and their adjacent normal tissues and made side-by-side comparison to reveal the negative correlation between SPOP and SUFU. The results showed that high immunoreactivity for SPOP and low immunoreactivity for SUFU were in the same ccRCC tissues comparing with their adjacent normal tissues (Supplementary Fig. S1B). In addition, analysis results for the Gene Expression Omnibus (GEO) database (GSE73731) also revealed a moderate negative correlation (*R* = −0.6) between SPOP and SUFU in ccRCC at mRNA level (Fig. 4E)^26^. Finally, Kaplan–Meier plotter tools were used to analyze the correlation between the mRNA levels of SUFU and the survival of patients with ccRCC by using publicly available datasets (http://kmplot.com/analysis/). Kapla–Meier survival curves and log rank test analyses demonstrated that ccRCC patients with low expression of SUFU had poorer relapse-free survival than those with high expression of SUFU (Fig. 4F). Taken together, our results characterized a negative correlation between SPOP and SUFU in ccRCC, and suggested a potential tumor suppressor role for SUFU in this disease.

### SUFU is a tumor suppressor in ccRCC

**Fig. 5:**
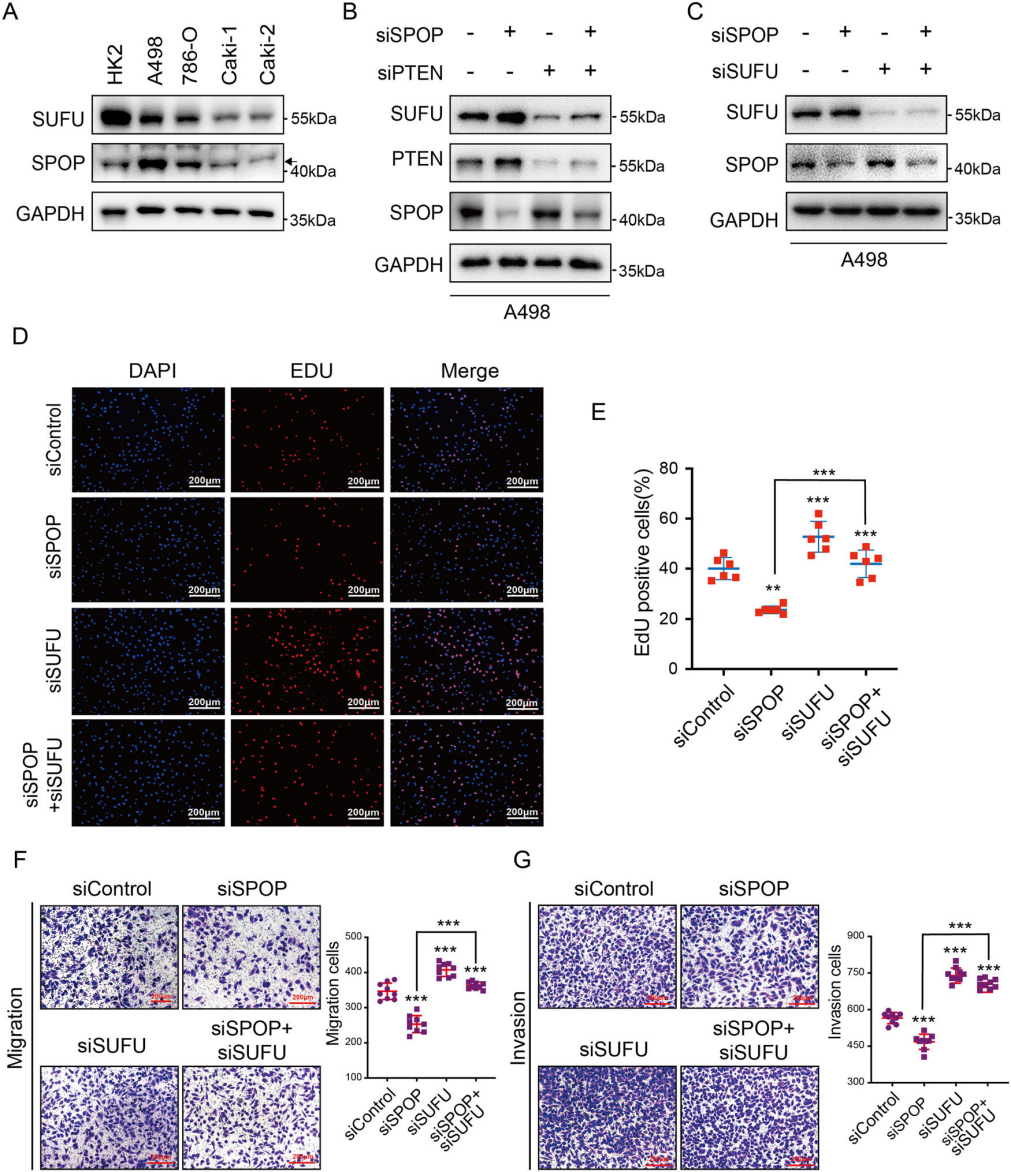
SPOP downregulates SUFU to enhance tumorigenesis and progression in ccRCC. **A** The relative abundances of SPOP and SUFU proteins were detected by western blotting in the normal kidney cells HK2 and four ccRCC cell lines, such as A498, 786-O, Caki-1, and Caki-2. **B** The protein levels of SUFU, PTEN, and SPOP were examined by western blotting in A498 cells transfected with indicated siRNA. **C** Western blot analysis showed the levels of SUFU in A498 cells transfected with *siSPOP* and *siSUFU*. **D**, **E** Fluorescence images and percentage quantification of EdU incorporation assays were shown. **F**, **G** Transwell assays and the quantification of migrated and invaded cells were applied to evaluate the migratory and invasive capacities of A498 cells expressing siRNA. **P < 0.01; ***P < 0.001; ns not significant (unpaired Student’s *t* test).

SUFU was first identified as a tumor suppressor in medulloblastoma^17^, however, whether SUFU can impede ccRCC tumorigenesis is not known. We found that the protein levels of SUFU detected in ccRCC cell lines, such as A498, 786-O, Caki-1, and Caki-2 were much less than in the normal kidney proximal tubular cell line HK2, while SPOP was highly expressed in A498 and 786-O cells, indicating that SUFU and SPOP were negatively correlated in ccRCC (Fig. 5A). Furthermore, SUFU was remarkable downregulated in SPOP-overexpressing A498 cells and upregulated in SPOP-knockdown cells (Fig. 1H, I). More importantly, when PTEN siRNA was co-transfected in ccRCC cells, SUFU was no longer increased in SPOP-knockdown cells, indicating that the protein level of SUFU could be regulated by SPOP–PTEN axis in ccRCC cell lines as well (Fig. 5B).

To investigate the biological function of SUFU in ccRCC, SUFU was silenced in HK2 and A498 cells, respectively (Supplementary Fig. S2A and Fig. 5C). SUFU knockdown promoted cell proliferation in the aspect of DNA synthesis according to 5-ethynyl-20-deoxyuridine (EdU) incorporation in both HK2 and A498 cells (Supplementary Fig. S2B, C and Fig. 5D, E). Furthermore, migratory and invasive capacities of HK2 and A498 cells were examined with transwell assays. As shown in Supplementary Fig. S2D, E and Fig. 5F, G, knockdown of SUFU significantly enhanced the migratory and invasive abilities of HK2 and A498 cells. These data indicated that SUFU, which is negatively regulated by SPOP, functions as a tumor suppressor in ccRCC.

### SPOP promotes tumorigenesis and progression through suppressing SUFU in ccRCC

SPOP acts a key regulatory hub in kidney cancer by degrading several tumor suppressors, including PTEN, which exhibits low levels in ccRCC patient samples, and SPOP-mediated degradation of PTEN promotes tumor cell proliferation in A498 ccRCC cells^5^. Previously, we found that SUFU was negatively regulated by SPOP–PTEN axis and acted as a tumor suppressor in ccRCC. In line with these results, whether SPOP–PTEN axis promotes tumorigenesis through downregulation of SUFU was further evaluated. To answer this question, SPOP and SUFU siRNA were co-transfected, and their knockdown efficiency was validated by western blotting (Fig. 5C). Proliferation was hampered by transfecting *SPOP* siRNA into A498 cells (Fig. 5D, E). Importantly, the cell growth defect induced by SPOP reduction depended on SUFU, as knockdown of SUFU could restore the proliferation, as indicated by EdU incorporation assays (Fig. 5D, E). Consistent with EdU results, SPOP-knockdown also reduced migratory and invasive abilities of A498 cells, which could be rescued by additional SUFU knockdown (Fig. 5F, G). These data indicated that SPOP–PTEN promotes tumorigenesis via suppressing SUFU in ccRCC.

### SPOP modulates SUFU repressor activity in SHH and WNT pathways in ccRCC

**Fig. 6:**
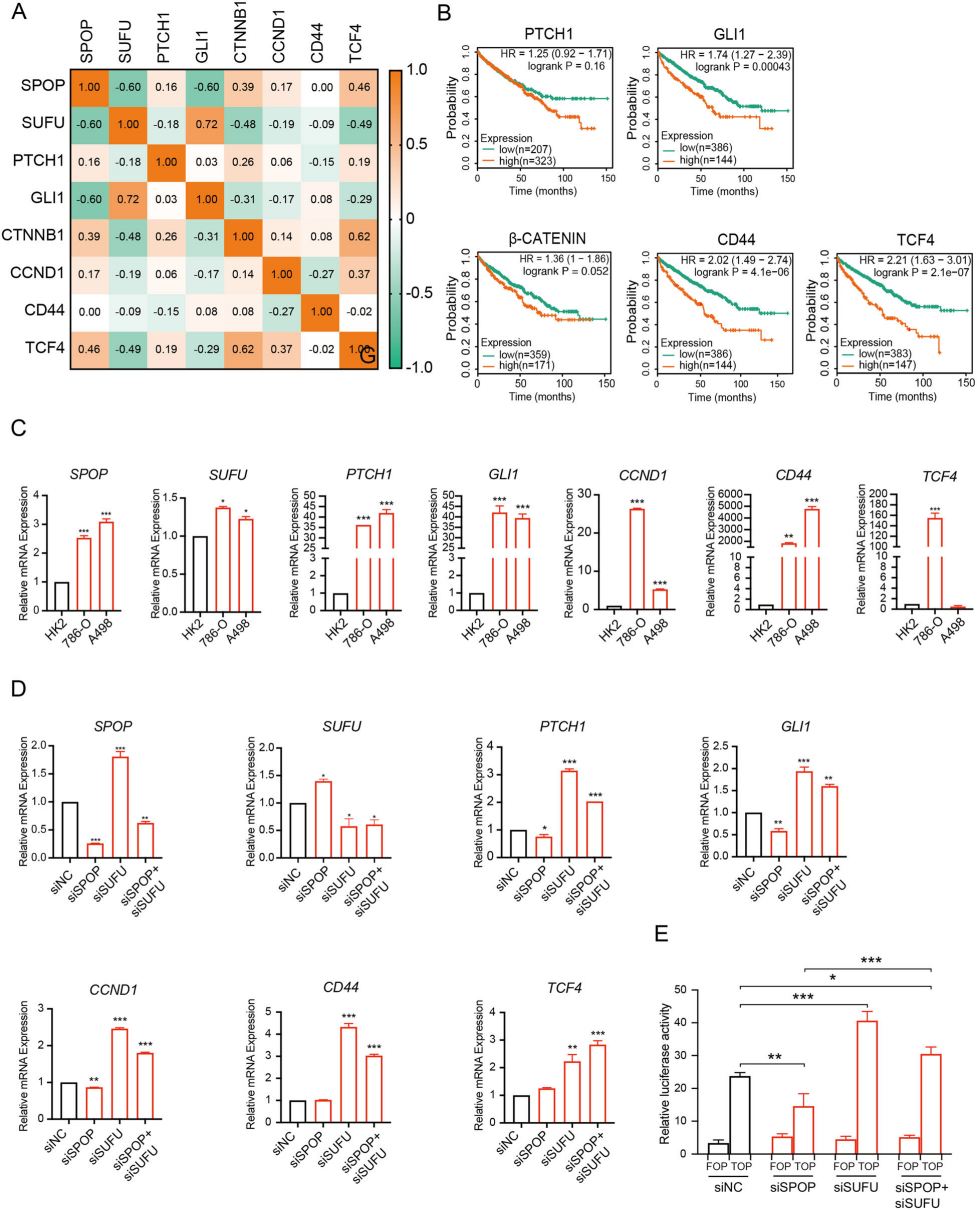
SPOP promotes tumor progression via activation of the SUFU-dependent SHH and WNT signaling pathways in ccRCC. **A** GEO analysis of 265 ccRCC samples (GSE73731) showed the correlations among the mRNA expression of SPOP, SUFU, and the target genes of SHH and WNT signaling pathways. **B** Kaplan–Meier analyses showed the associations between GLI1, PTCH1, β-CATENIN, CD44, and TCF4 expression and survival in patients with ccRCC. **C** qRT-PCR analysis was performed to evaluate the mRNA levels of *SPOP*, *SUFU*, and SHH target genes—*GLI1*, *PTCH1*, and WNT target genes—*CCND1*, *CD44*, *TCF4*. **D** Transcriptional levels of *SPOP*, *SUFU*, *GLI1*, *PTCH1*, *CCND1*, *CD44*, and *TCF4* were examined by qRT-PCR. **E** TOP-flash and FOP-flash reporter activities in controls cells and cells transfected with siRNA were shown. *P < 0.05; **P < 0.01; ***P < 0.001 (unpaired Student’s *t* test).

Given that SUFU is an important repressor of SHH and WNT signaling pathway, we considered whether SPOP promotes ccRCC tumorigenesis and progression by activating SUFU-dependent SHH and WNT signaling pathways. In keeping with this notion, we analyzed the mRNA expression of SPOP and target genes of SHH and WNT signaling, respectively, in a published ccRCC expression profile (GSE73731). As shown in Fig. 6A, Spearman correlation analysis revealed that the expression of SPOP was positively correlated with SHH target genes, such as Patched 1 (PTCH1), WNT transcription factor β-CATENIN, and target genes, such as cyclin D1 (CCND1) and transcription factor 4 (TCF4) by varying degrees. In addition, Kaplan–Meier analyses carried out by using the dataset from Kaplan–Meier plotter revealed that the patients with high levels of SHH targets (GLI1 and PTCH1) or WNT transcription factor (β-CATENIN) or WNT targets (CD44 and TCF4) all showed poorer survival than those with low expression, indicating that aberrantly activated SHH and WNT signaling indeed promote tumor progression in ccRCC (Fig. 6B).

Similarly, the transcriptional activities of SHH and WNT signaling were dramatically increased in both 786-O and A498 cells compared with HK2 cells, confirming that SHH and WNT signaling are dysregulated in ccRCC cells overexpressing SPOP (Fig. 6C). To further determine whether SPOP enhanced SHH and WNT signaling activities, and whether these changes were achieved by reducing SUFU in ccRCC, we knocked down SPOP, SUFU, or both of them in A498 cells, respectively. By quantifying the transcriptional responses by qRT-PCR, we demonstrated that knockdown of SPOP with siRNA could negatively modulate SHH and WNT signal transduction in A498 cells. As expected, knockdown of SUFU, which acts as the negative regulator of SHH and WNT signaling pathways, increased the transcriptional levels of target genes *GLI1*, *PTCH1*, *CD44*, *TCF4*, and *CCND1*. More importantly, SHH and WNT signaling activities suppressed by *siSPOP* could be restored or even further activated by knocking down SUFU simultaneously (Fig. 6D). To further evaluate the effect of this regulation on Wnt signaling, we also did TOP-flash assay. The results showed that knockdown of SPOP inhibited Wnt signaling as determined by TOP-flash activity, while its inhibitory effect could be suppressed by knocking down SUFU (Fig. 6E). Overall, our findings suggest that SPOP–SUFU axis promotes tumorigenesis and progression by activating SHH and WNT pathways in ccRCC.

### Inhibiting SHH and WNT has synergistic effects on suppressing ccRCC cell growth, migration, and invasion

**Fig. 7:**
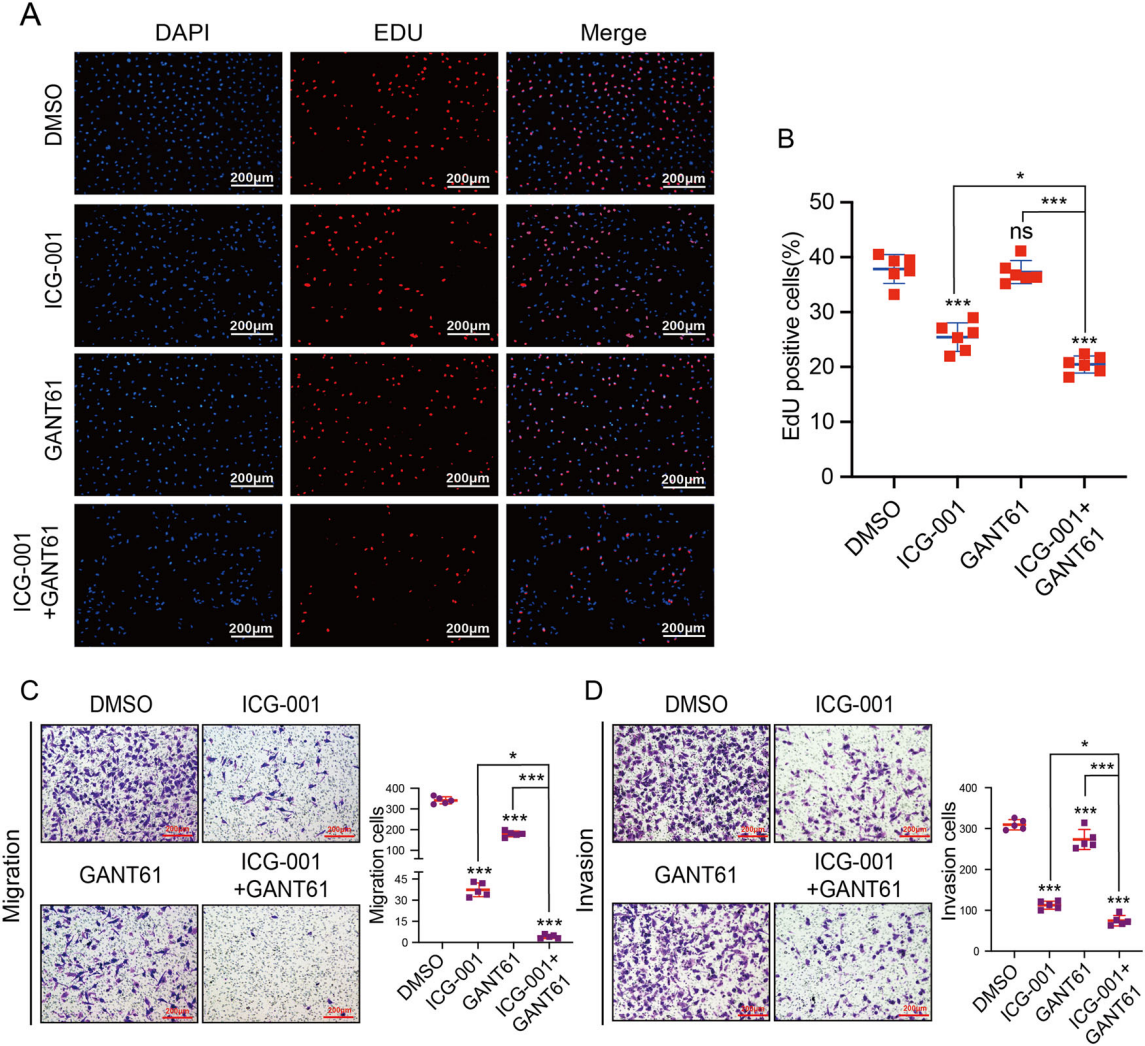
Combining SHH and WNT signaling pathway inhibitors is an effective treatment for ccRCC. **A**, **B** Fluorescence images and percentage quantification of EdU incorporation assays are shown. **C**, **D** Transwell assays and the quantification of migrated and invaded cells were applied to show the migratory and invasive capacities of A498 cells treated with inhibitors. *P < 0.05; **P < 0.01; ***P < 0.001; ns not significant (unpaired Student’s *t* test).

Since 2005, several agents targeting vascular endothelial growth factor and mTOR pathway have improved the overall survival of ccRCC patients^27^. Unfortunately, nearly all patients will develop resistance to targeted therapies, leading to a very low 5-year survival rate^3,27^. Therefore, more potent compounds that target specific signaling pathways involved in ccRCC pathogenesis are definitively needed to improve this poor prognosis. According to the above results, SPOP–PTEN–SUFU axis promotes tumorigenesis and progression by activating SHH and WNT pathways in ccRCC. To investigate whether simultaneous downregulation of SHH and WNT has a synergistic effect on suppressing ccRCC tumorigenesis and progression, we applied SHH inhibitor GANT61 and the WNT inhibitor ICG-001. To explore the effects of these two inhibitors on ccRCC cells, EdU incorporation assays were carried out to assess the proliferation capacity of the cells, whereas transwell assays were used to study migration and invasion. The EdU results revealed that the percentages of EdU-positive cells were reduced upon inhibitor treatment. Notably, single inhibitor treatment with ICG-001 was more effective than GANT61, whereas combinatorial treatment was the most effective approach (Fig. 7A, B). Consistently with the EdU incorporation assays, transwell assays showed that treatment with ICG-001 more effectively inhibited migration and invasion than treatment with GANT61, whereas combinatorial treatment had synergistic effects (Fig. 7C, D). These data demonstrated that inhibiting SHH and WNT signaling produces synergistic effects on suppressing ccRCC cell proliferation, migration, and invasion, providing new rationales for novel combinational therapies using two signaling inhibitors.

## Discussion

As a ubiquitin E3 ligase, SPOP degrades different substrates in different tumors, and thus plays different roles in tumor development^28–30^. For example, in prostate cancer, SPOP, acting as a tumor suppressor, inhibits the development of prostate cancer by degrading carcinogenic factors androgen receptor^31^, c-MYC^32^, ETS-related gene^33^, and Egl-9 family hypoxia-inducible factor 2 (ref. ^34^). Conversely, some studies have demonstrated that hypoxia leads to cytoplasmic accumulation of SPOP, which promotes tumorigenesis through the ubiquitination and degradation several tumor suppressors, such as PTEN, indicating that SPOP acts as a cancer-promoting factor in ccRCC^5^. Our results also showed that SPOP was highly expressed in most ccRCC clinical tissues and ccRCC cell lines, and the knockdown of *SPOP* in A498 cells suppressed ccRCC tumorigenesis and progression by reducing cancer cell growth, migration, and invasion. Importantly, we found SHH and WNT signaling pathway activities were reduced with SPOP downregulation, suggesting that the tumor-promoting function of SPOP stands in activation of the SHH and WNT signaling pathways, which have been shown to be aberrantly activated in ccRCC^35,36^.

SHH signaling pathway plays key roles in embryonic development and adult tissue homeostasis^37^. Previous studies have revealed that SHH signaling is reactivated in many types of human malignancy, including ccRCC^38,39^. Elevated expression of SHH receptor Smoothened (SMO) and transcription factor GLI1 can enhance ccRCC cell proliferation, whereas SMO inhibitor cyclopamine can suppress tumor growth by decreasing cell proliferation and inducing cell apoptosis in ccRCC in vitro and in vivo^38^. In line with those studies, our results demonstrated that SHH signaling was highly activated in ccRCC cells. In a database analysis, the patients with higher expression of SHH target genes, such as GLI1 and PTCH1, had a poorer prognosis than those with lower expression. Another pathway identified in our study was WNT signaling pathway, which also plays critical roles in embryonic development and carcinogenesis. Dysregulated WNT signaling caused by abnormally activated β-CATENIN was also found in ccRCC patients with advanced cancer and associated with a decrease in survival, and WNT inhibitor ICG-001 was shown to impair tumor growth in several experimental models, such as sphere, organoid culture, and patient-derived xenografts (PDXs)^40,41^. Here, we further confirmed WNT upregulation in ccRCC cells by showing the elevated mRNA levels of downstream targets, such as *CCND1*, *CD44*, and *TCF4*. It is worth noting that combinations of SHH and WNT inhibitors have strong suppressive effects on cell proliferation, migration, and invasion in ccRCC. Considering the limited efficacy and drug resistance of tyrosine kinase inhibitors, including sunitinib and sorafenib, that are now approved and in clinical used for advanced or metastatic ccRCC treatmnet^42^, our findings provide a promising therapeutic approach, combinatorial regulation of SHH and WNT affected by SPOP may have the potential to be a more effective ccRCC treatment.

**Fig. 8:**
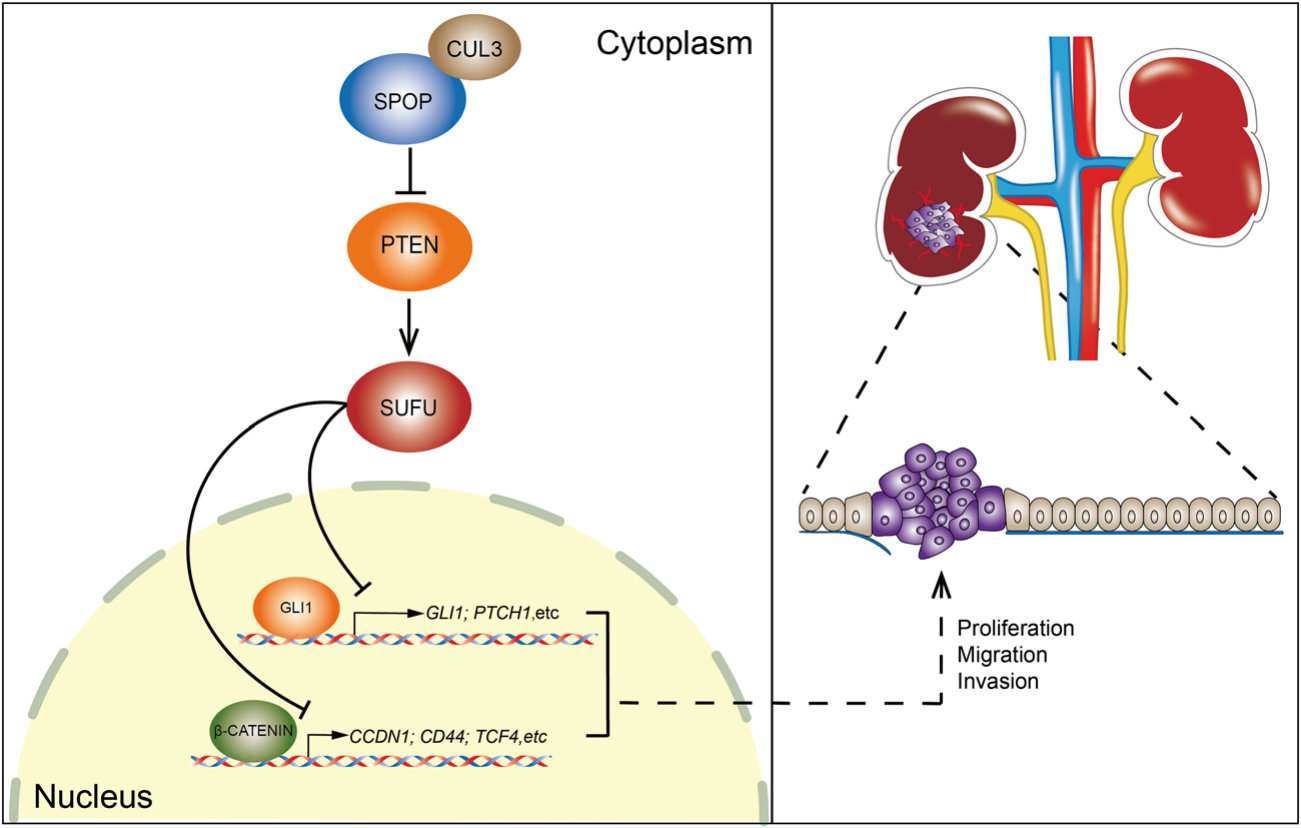
A model shows that SPOP downregulates SUFU through PTEN in ccRCC. SPOP–PTEN axis promotes tumor progression by modulating SUFU repressor activity in SHH and WNT pathways.

Then, we investigated the molecular mechanism underlying SPOP-mediated activation of SHH and WNT signaling in ccRCC. Notably, SPOP is reported to degrade GLI2 which functions mostly as an activator in SHH pathway^5,43^. This degradation seemed inconsistent with the upregulation of SHH-GLI1 signaling found in ccRCC^44^, therefore, we had reason to suspect that other regulators of SHH pathway might be suppressed by SPOP. Furthermore, SPOP enhances β-CATENIN nuclear translocation to promote invasiveness in ccRCC cells, but the specific mechanism remains unknown^7^. Fortunately, we found that SUFU which has been reported to be a cross-linking point for SHH and WNT pathways, was downregulated by SPOP in ccRCC^45^. Previous studies have illustrated that SUFU mainly acts as a negative regulator in SHH and WNT pathways, suppressing the functions of GLI and β-CATENIN partially by regulating their cellular localization^11,15^. Loss of SUFU has been shown to promote tumorigenesis and progression in many cancers in mammals. Unfortunately, the impact of such an interesting gene on kidney cancer has not yet been systematically studied. In our study, we first identified SUFU as a tumor suppressor in ccRCC through the use of bioinformatics analysis and experimental verification. Corresponding to the increase in SPOP expression and the decrease in PTEN expression in ccRCC tissues^5^, it was observed a lower expression of SUFU in clinical samples and cell lines. Furthermore, SPOP was found to reduce SUFU through PTEN, which is degraded by this E3 ligase in ccRCC cells. Importantly, knockdown of SUFU could restore the hampered proliferation and aggressiveness of A498 cells mediated by SPOP siRNA due to aberrant activation of SHH and WNT pathways, suggesting that SPOP–PTEN promotes tumorigenesis and progression in ccRCC partially by upregulating the activities of the SUFU-dependent SHH and WNT pathways (Fig. 8). In addition, we also observed a negative correlation between SPOP and SUFU accompanied by dysregulated SHH signaling in ovarian cancer (Figs. 1J, K and 4A, B, and Supplementary Fig. S3A, B). Further investigation is required to discover the function of SUFU downregulation by SPOP in ovarian cancer.

In this study, we demonstrate that SPOP downregulates SUFU in mammals. Interestingly, although this downregulation depends on cullin-3(Cul3)-SPOP E3 ligase, SUFU is not a direct substrate of SPOP. Further studies explore that SPOP-mediated reduction of SUFU relies on PTEN, which is degraded by SPOP in ccRCC cells. Importantly, knockdown of SPOP reduces ccRCC cell ability of proliferation, migration, and invasion, which can be rescued through additional knockdown of SUFU as the result of elevated activities of SHH and WNT pathways, indicating that SPOP promotes tumorigenesis and progression via activating SUFU-dependent SHH and WNT signaling pathway. Combination of these two pathways inhibitors shows an enhanced effect in suppressing cell proliferation and aggressiveness, which remains to be further studied in 3D tumor organoids and PDXs in mice to overcome the limitation imposed by 2D cell model system.

## Materials and methods

### Cell culture

*Spop*-knockout MEF was a gift from Dr. Jun Yan (Model Animal Research Center of Nanjing University, China). Human cell lines were purchased from ATCC and maintained with 1% penicillin and streptomycin (Gibco Life Technologies, 15140122) at 37 °C with 5% CO_2_. A498 cells were cultured in MEM/EBSS medium (HyClone, SH30024.01) supplemented with 10% fetal bovine serum (FBS, Gibco, 10100154) and 2× nonessential amino acids (Gibco,11140050). HK2, MEF, and 786-O cells were cultured in DMEM/F12 medium (HyClone) supplemented with 10% FBS (Gibco). Caki-1, Caki-2, and SKOV3 cells were cultured in McCoy’s 5A medium (KeyGENE BioTECH). 293T cells were cultured in DMEM (Gibco,12100046) supplemented with 10% FBS (Wisent, 085-150), 1× glutamine (Gibco, 35050061), and 1 mM sodium pyruvate (Gibco, 11360070).

### Plasmids

The cDNA sequences of human CUL3, SPOP, PTEN, and SUFU were amplified from 293T cells. Flag-tagged SPOP, Myc-tagged CUL3, PTEN, and SUFU were cloned into pRK5 vector, according to the manufacturer’s instructions (Vazyme, C112-01). Flag-tagged SPOP-Y87C, SPOP-W131G, Myc-tagged CUL3-Y62G, and CUL3-K711S were generated, according to the manufacturer’s protocols (Vazyme, C214-01). Flag-tagged SPOP fragments (MATH, BTB, and Δ3-box) were amplified by PCR, using pRK5-Flag-SPOP as the template and constructed into RK5 vector.

### siRNAs

siRNAs were designed and purchased from Genepharma (Shanghai, China). The sequences of siRNAs were listed in Supplementary Table S2.

### Transfection

Cells were transfected with siRNA using Lipofectamine RNAiMAX (Invitrogen), or with plasmids using GBfectene-Elite (Genebank Bioscience lnc), following the manufacturer’s instructions. Cells were harvested after 48 h transfection for studies.

### RNA extraction and quantitative real-time PCR analyses

Total RNA was isolated from cultured cells using the RNAiso Plus reagent (TakaRa), and reverse transcription (RT) experiment was carried out using the HiScript II Q RT SuperMix for qRT-PCR kit (Vazyme). RT-PCR was carried out using the AceQ qPCR SYBR Green Master Mix (Vazyme) on a RT-PCR system (Roche) with primers as listed in Supplementary Table S3. The expression levels of indicated genes were normalized to an internal control(18 S), and the relative expression levels were evaluated using the 2^−ΔΔCT^ method. Each target was measured in triplicate.

### Western blotting

The indicated cells were washed twice with cold PBS and subsequently lysed in RIPA lysis buffer (150 mM NaCl, 50 mM Tris-HC, pH 7.5, 1 mM EDTA, pH 8.0, 0.5% sodium deoxycholate, 1% NP-40, 0.1% SDS, 2% sodium fluoride, and 0.5% sodium orthovanadate supplemented with protease inhibitor cocktail) at 4 °C for 30 min. After centrifugation to remove debris (14,000 × *g*, 20 min), the protein concentration of each cell lysate sample was determined by the bicinchoninic acid assay. Each lysate was denatured in loading buffer at 95 °C for 5 min. Then, the lysates were resolved by 8% SDS–PAGE and transferred onto PVDF membranes. The membranes were blocked with 5% nonfat milk in TBST and probed with indicated primary antibodies followed by horseradish peroxidase-conjugated secondary antibodies (Jackson ImmunoResearch). Signals were visualized using Clarity Western ECL substrate (Bio-Rad). Densitometric analysis was carried out using ImageJ image analysis software.

### Inhibitor assays

Proteasome inhibitor MG132 was added to 293T cells at a final concentration of 20 μM for 4 h before cell harvest. After treatment with 10 μM BPV for 24 h, 293T cells were harvested for western blot analysis. A498 cells were treated with ICG-001 (20 μM), GANT61(20 μM), or their combinations (ICG-001 and GANT61) for 5 days. Then, cells were used for EdU incorporation, transwell migration, and Matrigel invasion assays.

### Protein turnover assay

To measure protein turnover of endogenous SUFU, 293T cells transfected with RK5-Flag-SPOP or vector control were treated with CHX (10 μM; Sigma) to block protein synthesis. At each time point, the cells were lysed in RIPA buffer for western blot analysis.

### Coimmunoprecipitation

Transfected 293T cells were lysed in RIPA lysis buffer at 4 °C for 30 min, and the lysates were centrifugated for 20 min at 14,000 × *g* to remove debris. After measuring the protein concentration of each cell lysate sample, immunoprecipitation was carried out with anti-FLAG M2 affinity gel (1 μL/1500 μg protein, Sigma) and anti-Myc antibody (1 μL/300 μg protein, Thermo Fisher) coupled to Dynabeads Protein G (Thermo Fisher), and the isolated proteins were used for western blot analysis.

### TOP-flash assay

Luciferase assays with cells were carried out essentially as described^46^. A498 cells were seeded in 24-well plates and transfected with the Wnt/β-catenin signaling reporter TOP-flash/FOP-flash reporter plasmids with Renilla control using FuGENE HD. After 6–8 h, indicated siRNAs were transfected into the cells using lipofectamine RNAiMAX for 48 h. Luciferase activities from both reporters were measured using the Dual Luciferase Reporter Assay Kit (Vazyme), following the manufacturer’s instructions.

### Tissue samples and immunohistochemistry (IHC)

To confirm SPOP and SUFU protein expression in human cancers, a multi-organ cancer tissues microassay (BCN963a) containing 16 kinds of human cancers was examined by IHC. Then, another tissue microassay (KD1504) was carried out to detect SPOP and SUFU protein levels in ccRCC, which includes 50 ccRCC and adjacent noncancerous kidney tissues. All the microchips were obtained from Alenabio (Xi’an, China). The staining was evaluated by scanning the section under low magnification and confirmed under high magnification. All stains were assessed according to the histologic scoring system (H-score) based on the product of staining intensity (negative, low, median, and high). Each section was scored independently by two pathologists and a third pathologist determined the final score if there was any inconsistency.

### EdU incorporation assay

Cells were seeded at 4 × 10^4^ cells/well in 24-well plates containing round coverslips and maintained in medium overnight. Cell proliferation was further evaluated through measuring the incorporation of EdU with EdU Cell Proliferation Assay Kit (RiboBio). Images were captured by using a fluorescence microscope, and the proliferating cells in five different fields were counted.

### Transwell migration and Matrigel invasion assay

The 24-well plate with 8 µm pore polycarbonate membrane inserts (Millipore) was used to analyze the invasive and migratory abilities of tumor cells. For the migration assay, after adding 600 μL 10% FBS medium into the lower chambers, 3.6 × 10^4^ cells in 300 μL serum-free medium were seeded into the insert for incubation at 37 °C in 5% (v/v) CO_2_ incubator for 8 h. Then, the cells migrating to the lower surface of the membrane insert were stained with the crystal violet (Beyotime, C0121) and quantified by counting five randomly chosen microscopic fields. For the invasion assay, the membrane was coated with 60 μL diluted Matrigel (1:50; Corning) and 3.6 × 10^4^ cells in 300 μL serum-free medium were added. Simultaneously, 600 μL 10% FBS medium was added to the lower chambers, and the plate was incubated at 37 °C in a 5% (v/v) CO_2_ incubator for 12–16 h. Then, cells invading into the lower surface of the membrane insert were stained with crystal violet and quantified by counting five randomly chosen microscopic fields.

### Analysis of published datasets

Human ccRCC expression dataset from GSE73731 was used. Correlations between different gene were determined by Spearman correlation analysis. Overall survival curves were analyzed with Kaplan–Meier plotter (http://kmplot.com/analysis/index.php?p=service&Cancer=pancancer_rnaseq). The best cutoff value was autoselected in the analysis. A total of 530 ccRCC samples were divided into high and low groups, according to the cutoff value. The hazard ratio with 95% confidence interval and log rank *P* value were calculate, and significance was set at *P* < 0.05.

### Statistical analysis

qRT-PCR, EdU, migration, and invasion data were represented as mean ± SD. Statistical significance was determined by unpaired Student’s *t* test, one-way ANOVA, or two-way ANOVA with comparison to the control group. Each experiment was repeated at least three times. *P* value < 0.05 was considered statistically significant.

### Key resources and reagents information

Further information for key resources and reagents was listed in Supplementary Table S1.

## Supplementary information

SUPPLEMENTAL MATERIAL

Supplemental Figure1

Supplemental Figure2

Supplemental Figure3

Supplementary Figure Legends
