# Supplementary material for: SPOP–PTEN–SUFU axis promotes progression of clear cell renal cell carcinoma via activating SHH and WNT pathway
Source: Cell Death Discov. 2021 May 21;7:120. doi: 10.1038/s41420-021-00484-2 (PMC8140158; doi:10.1038/s41420-021-00484-2)
Supplement: Supplementary file 1 — SUPPLEMENTAL MATERIAL [file 41420_2021_484_MOESM1_ESM.docx]

SUPPLEMENTAL INFORMATION

SPOP-PTEN-SUFU axis promotes progression of clear cell renal cell carcinoma via activating SHH and WNT pathway

Bo’ang Han, Zhen Sun, Tingting Yu, Yu Wang, Lun Kuang, Tianyuan Li, Jing Cai, Qing Cao, Yuan Xu, Binbin Gao, Steven Y Cheng, Shen Yue and Chen Liu

Table S1: Key resources and reagents;

Table S2: siRNAs sequences;

Table S3: The qRT-PCR primers;

Table S1. resources and reagents

| REAGENT or RESOURCE | SOURCE | IDENTIFIER | APPLICATION |
| --- | --- | --- | --- |
| Antibodies | | | |
| Rabbit anti GLI1 | CST, USA | Cat# 2534 | WB(1:1000) |
| Mouse anti Akt1 | CST, USA | Cat# 2967 | WB(1:1000) |
| Mouse anti Phospho-Akt | CST, USA | Cat# 4051 | WB(1:1000) |
| Goat anti GLI2 | NOVUS, USA | Cat# NB600-874 | WB(1:1000) |
| Goat anti GLI3 | R&D, USA | Cat# AF3690 | WB(1:1000) |
| Rabbit anti β-CATENIN | Proteintech, China | Cat# 51067-2-AP | WB(1:1000) |
| Rabbit anti SUFU | Proteintech, China | Cat# 26759-1-AP | WB(1:1000),  IHC(1:200) |
| Rabbit anti PTEN | CST, USA | Cat# 9188 | WB(1:1000) |
| Rabbit anti SPOP | Proteintech, China | Cat# 16750-1-AP | WB(1:1000), IHC(1:300) |
| Rabbit anti GAPDH | Santa Cruz Biotechnology, USA | Cat# sc-32233 | WB(1:1000) |
| Mouse anti Flag | Sigma, USA | Cat# F1804 | WB(1:1000) |
| Mouse anti Myc | Sigma,USA | Cat# sc-40 | WB(1:1000), IP(1μL/300μg) |
| Mouse anti HA | Thermo Scientific, USA | Cat# 26183 | WB(1:1000) |
| anti Mouse | Jackson ImmunoReasearch | Cat# 115-035-003 | WB(1:5000) |
| anti Rabbit | Jackson ImmunoReasearch | Cat# 111-005-003 | WB(1:5000) |
| anti Goat | Jackson ImmunoReasearch | Cat# 705-035-147 | WB(1:5000) |
| Chemicals | | | |
| TRIzol | Takara, Japan | Cat# 9109 | RNA Extraction |
| Complete protease inhibitor cocktail | Roche, USA | Cat# 04693132001 | Protease inhibitor |
| Bisperoxovanadium(HOpic,BPV) | Selleck, USA | Cat# S8651 | PTEN inhibitor |
| ICG-001 | MCE, USA | Cat# HY-14428 | β-catenin/TCF inhibitor |
| GANT61 | Sigma, USA | Cat# G9048 | Hedgehog/Gli1 inhibitor |
| Cycloheximide (CHX) | Millipore, USA | Cat# 66-81-9 | Protein synthesis inhibitor |
| Crystal Violet | Beoytime, China | Cat# C0121 | Staining |
| Lipofectamine™ RNAiMAX Transfection Reagent | Invitrogen,USA | Cat# 13778150 | Transfect siRNA |
| FuGENE HD | Invitrogen, USA | Cat# E2311 | Transfect Plasmid |
| Matrigel | Corning, USA | Cat# 356234 | Cell Motility |
| Critical Commercial Sources | | | |
| HiScript II Q RT SuperMix for qPCR kit | Vazyme, China | Cat# R223-01 | RT-PCR |
| AceQ qPCR SYBR Green Master Mix | Vazyme, China | Cat# Q111-02 | Real-time PCR |
| Bicinchoninic acid (BCA) assay | Thermo Scientific, USA | Cat# 23225 | Protein quantitative |
| ClonExpress II One Step Cloning Kit | Vazyme, China | Cat# Q112-01 | Gene Clone |
| Mut Express II Fast Mutagenesis Kit V2 | Vazyme, China | Cat# Q214-01 | Mutagenesis |
| Dual Luciferase Reporter Assay Kit | Vazyme,China | Cat# DL101-01 | Dual Luciferase Reporter Assay |
| Mouse anti Flag M2 affinity Gel | Sigma,USA | Cat# A2220 | IP |
| EdU Cell Proliferation Assay Kit | RiboBio,China | Cat# C10310-1 | Cell Proliferation |
| SAP Immunohistochemistry Assay Kit (Mouse & Rabbit) | ZSGB-BIO,China | Cat# SAP-9100 | IHC |
| Tissue Samples | | | |
| Multi-organ cancer tissues microassay | Alenabio,China | Cat# BCN963a | IHC |
| Human renal cell carcinoma tissues microassay | Alenabio,China | Cat# KD1504 | IHC |

Table S2. siRNA sequences

| **siRNA** | **Sequence** | |
| --- | --- | --- |
|  | **sense（5'-3'）** | **antisense（5'-3'）** |
| siControl | UUCUCCGAACGUGUCACGUTT | ACGUGACACGUUCGGAGAATT |
| siSPOP | GAGAGUCAACGGGCAUAUATT | UAUAUGCCCGUUGACUCUCTT |
| siSUFU | GGAGAGGACUCGAGAUCAATT | UUGAUCUCGAGUCCUCUCCTT |
| siPTEN | GGUGUAAUGAUAUGUGCAUTT | GGUGUAAUGAUAUGUGCAUTT |

Table S3. qRT-PCR primers

| **Gene** | **Sequence** | |
| --- | --- | --- |
|  |  |  |
|  | **sense（5'-3'）** | **antisense（5'-3'）** |
| GLI1 | AGCTAGAGTCCAGAGGTTCAA | TAGACAGAGGTTGGGAGGTAAG |
| SUFU | ACATGCTGCTGACAGAGGAC | CACTGCTGGGCTGAGTGTAG |
| PTEN | GACCAGAGACAAAAAGGGAGTA | ACAAACTGAGGATTGCAAGTTC |
| SPOP | GCCCTCTGCAGTAACCTGTC | GTCTCCAAGACATCCGAAGC |
| PTCH1 | GGGTGGCACAGTCAAGAACAG | TACCCCTTGAAGTGCTCGTACA |
| CD44 | TCTGAATCAGATGGACACTCAC | CATTGCCACTGTTGATCACTAG |
| TCF4 | ATAATGACGATGAGGACCTGAC | GGATTCAGATTCCTTTCTCGGA |
| CCND1 | CCTGGCATATCTCATCGAGATT | TCTTCATCTCCTGTTGGTTACC |
| GAPDH | CGACCACTTTGTCAAGCTCA | CCCTGTTGCTGTAGCCAAAT |
