# Supplementary figures and images for: SPOP–PTEN–SUFU axis promotes progression of clear cell renal cell carcinoma via activating SHH and WNT pathway
Source: Cell Death Discov. 2021 May 21;7:120. doi: 10.1038/s41420-021-00484-2 (PMC8140158; doi:10.1038/s41420-021-00484-2)

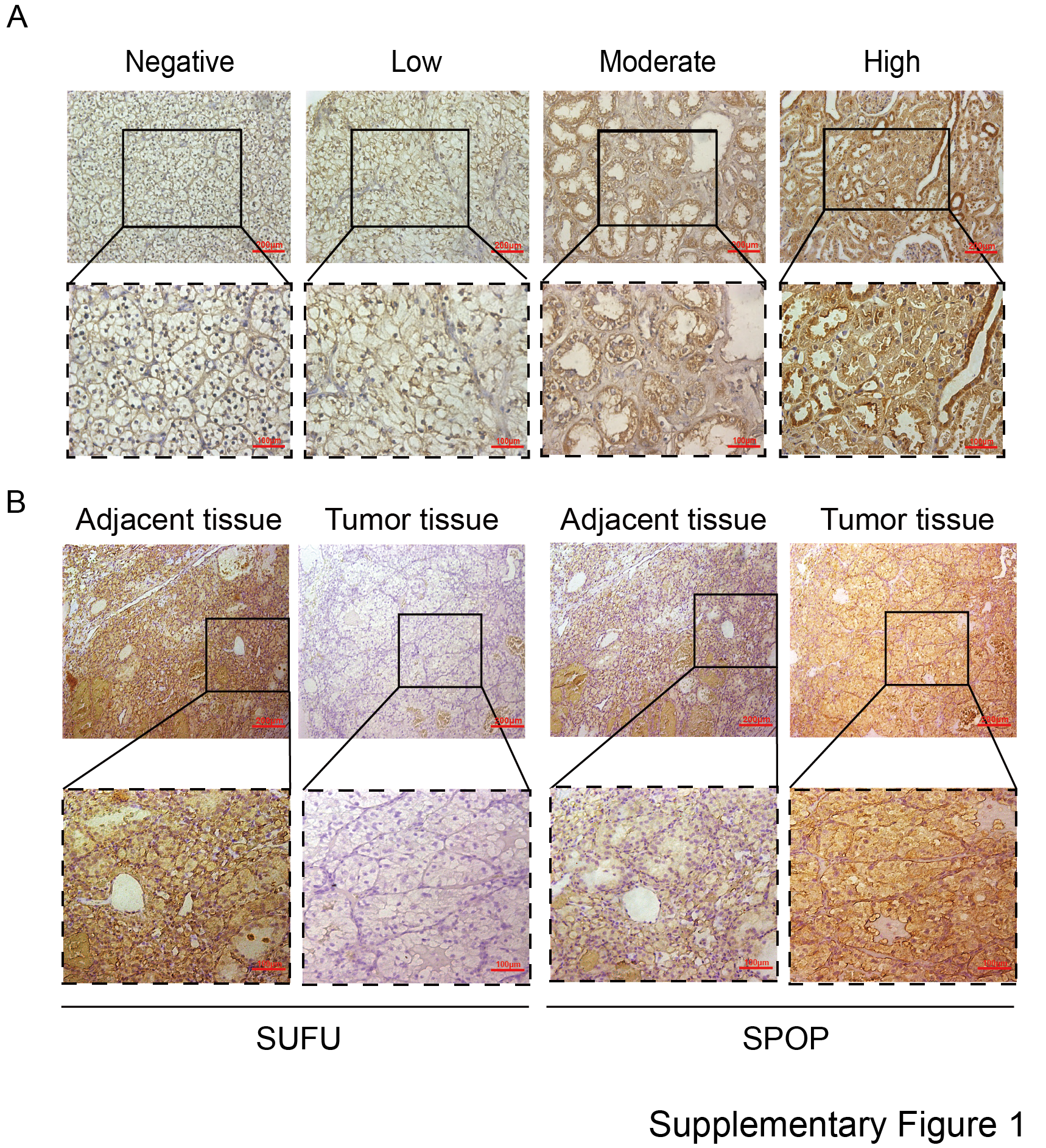

Supplement: Supplementary file 2 — Supplemental Figure1 [file 41420_2021_484_MOESM2_ESM.png]

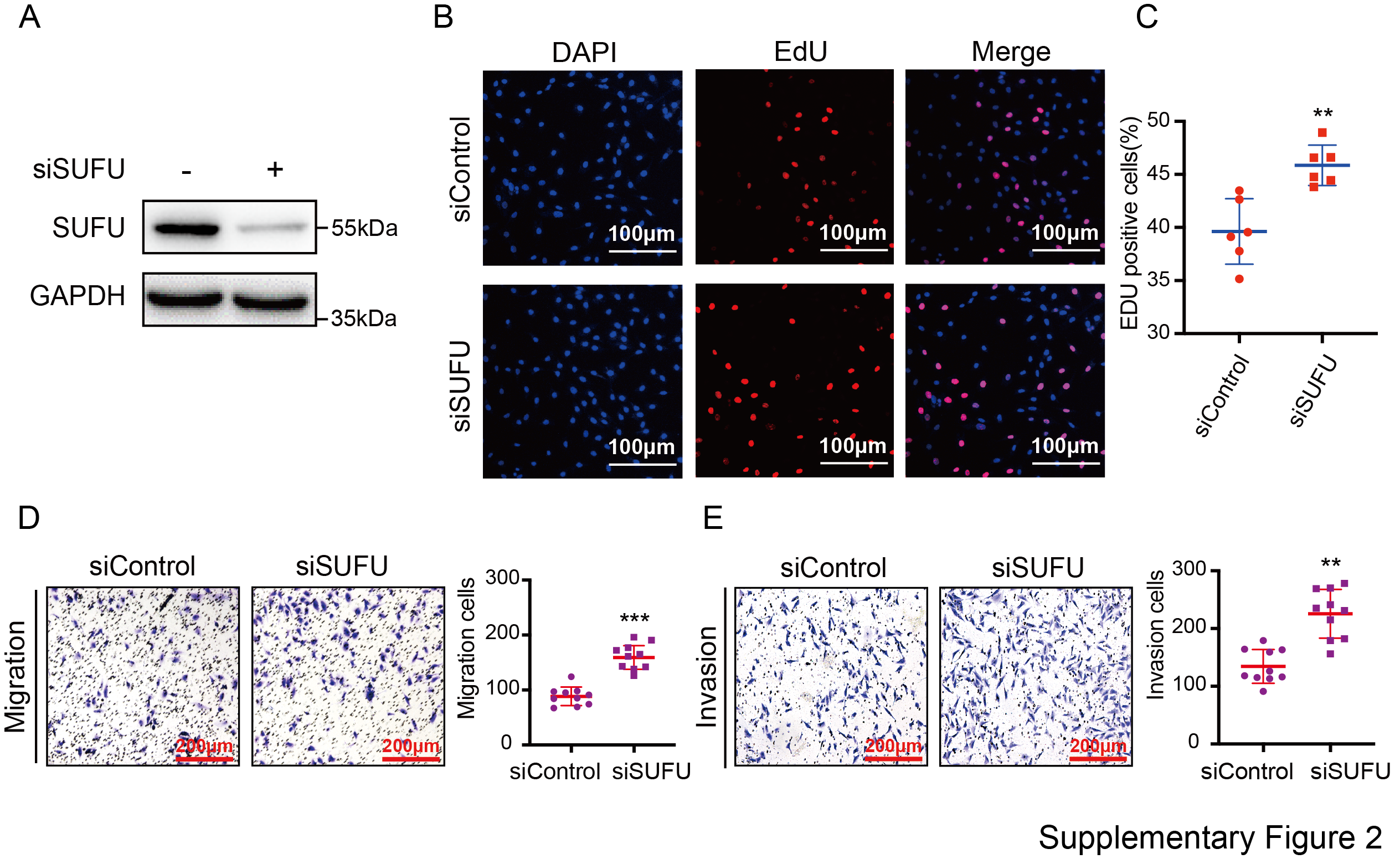

Supplement: Supplementary file 3 — Supplemental Figure2 [file 41420_2021_484_MOESM3_ESM.png]

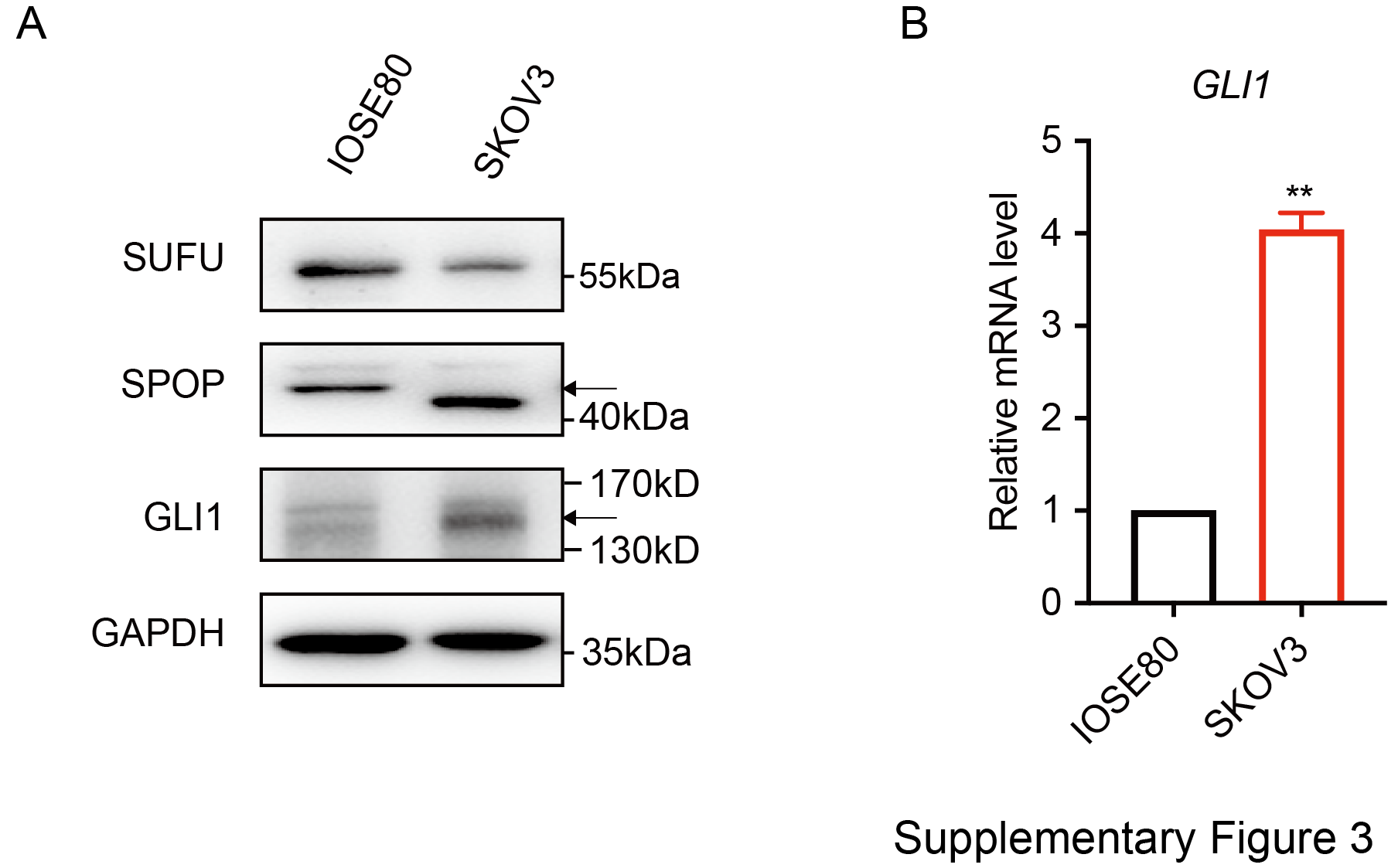

Supplement: Supplementary file 4 — Supplemental Figure3 [file 41420_2021_484_MOESM4_ESM.png]
