# Supplementary material for: SPOP–PTEN–SUFU axis promotes progression of clear cell renal cell carcinoma via activating SHH and WNT pathway
Source: Cell Death Discov. 2021 May 21;7:120. doi: 10.1038/s41420-021-00484-2 (PMC8140158; doi:10.1038/s41420-021-00484-2)
Supplement: Supplementary file 5 — Supplementary Figure Legends [file 41420_2021_484_MOESM5_ESM.docx]

SUPPLEMENTAL INFORMATION

SPOP-PTEN-SUFU axis promotes progression of clear cell renal cell carcinoma via activating SHH and WNT pathway

Bo’ang Han, Zhen Sun, Tingting Yu, Yu Wang, Lun Kuang, Tianyuan Li, Jing Cai, Qing Cao, Yuan Xu, Binbin Gao, Steven Y Cheng, Shen Yue and Chen Liu

Supplementary Figure Legends

**Fig. S1 SUFU is negatively correlated with SPOP in ccRCC tissues. A** The images for negative, low, moderate and high IHC expression were shown. **B** Representative IHC staining for SUFU and SPOP were shown in the same ccRCC tissues and normal adjacent tissues.

**Fig. S2 SUFU inhibited cell growth and aggressiveness in HK2. A** The relative abundance of SUFU protein were detected by western blot in the normal kidney cells HK2 transfected with *SUFU* siRNA or its non-targeted control. **B,C** Fluorescence images and percentage quantification of EdU incorporation assays are shown. **D** Cell migration were examined when HK2 cells were transfected by indicated siRNA. **E** Transwell assays and the quantifications of invasion cells were applied to show the invasion capacity of HK2 cells expressing siRNA. (**) P < 0.01; (***) P < 0.001 (unpaired Student’s t-test).

**Fig. S3 SPOP and SUFU showed a negative correlation accompanied with an aberrant activated SHH in ovary cancer. A** The protein levels of SPOP and SUFU were measured by western blot in IOSE80 and SKOV3 cells. **B** The mRNA level of SHH target gene GLI1 was examined by qRT-PCR. (**) P < 0.01 (unpaired Student’s t-test).
